# Thermo-responsive Fluorescent Nanoparticles for Multimodal Imaging and Treatment of Cancers

**DOI:** 10.7150/ntno.39810

**Published:** 2020-01-01

**Authors:** Nikhil Pandey, Jyothi U. Menon, Masaya Takahashi, Jer-Tsong Hsieh, Jian Yang, Kytai T. Nguyen, Aniket S. Wadajkar

**Affiliations:** 1 Department of Bioengineering, University of Texas at Arlington, Arlington, TX 76019, USA; 2 Department of Biomedical Engineering, University of Texas Southwestern Medical Center, Dallas, TX 75390, USA; 3 Department of Biomedical and Pharmaceutical Sciences, College of Pharmacy, University of Rhode Island, Kingston, RI 02881, USA; 4 Department of Radiology, University of Texas Southwestern Medical Center, Dallas, TX 75390, USA; 5 Department of Urology, University of Texas Southwestern Medical Center, Dallas, TX 75390, USA; 6 Department of Biomedical Engineering, Pennsylvania State University, University Park, PA 16802, USA; 7 Department of Neurosurgery, University of Maryland School of Medicine, Baltimore, MD 21201, USA; 8 Marlene and Stewart Greenebaum Comprehensive Cancer Center, University of Maryland School of Medicine, Baltimore, MD 21201, USA

**Keywords:** theranostic systems, thermo-responsive polymers, photoluminescent polymers, solid tumors, magnetic nanoparticles

## Abstract

Theranostic systems capable of delivering imaging and therapeutic agents at a specific target are the focus of intense research efforts in drug delivery. To overcome non-degradability and toxicity concerns of conventional theranostic systems, we formulated a novel thermo-responsive fluorescent polymer (TFP) and conjugated it on the surface of iron oxide magnetic nanoparticles (MNPs) for imaging and therapeutic applications in solid tumors. Methods: TFP-MNPs were synthesized by copolymerizing poly(*N*-isopropylacrylamide), allylamine and a biodegradable photoluminescent polymer, and conjugating it on MNPs via a free radical polymerization reaction. Physicochemical properties of the nanoparticles were characterized using Fourier transform infrared spectroscopy, dynamic light scattering, and vibrational sample magnetometry. Nanoparticle cytocompatibility, cellular uptake and cytotoxicity were evaluated using *in vitro* cell assays. Finally, *in viv*o imaging and therapeutic efficacy studies were performed in subcutaneous tumor xenograft mouse models. Results: TFP-MNPs of ~135 nm diameter and -31 mV ζ potential maintained colloidal stability and superparamagnetic properties. The TFP shell was thermo-responsive, fluorescent, degradable, and released doxorubicin in response to temperature changes. *In vitro* cell studies showed that TFP-MNPs were compatible to human dermal fibroblasts and prostate epithelial cells. These nanoparticles were also taken up by prostate and skin cancer cells in a dose-dependent manner and exhibited enhanced killing of tumor cells at 41°C. Preliminary *in vivo* studies showed theranostic capabilities of the nanoparticles with bright fluorescence, MRI signal, and therapeutic efficacy under magnetic targeting after systemic administration in tumor bearing mice. Conclusion: These results indicate the potential of TFP-MNPs as multifunctional theranostic nanoparticles for various biological applications, including solid cancer management.

## Introduction

Theranostic drug delivery systems are attractive platforms comprising diagnostic and therapeutic agents for simultaneous imaging and therapy in a single setting. Theranostic systems can potentially assist in non-invasive monitoring of both the disease progression and nanoparticle drug delivery route 1. Stimuli-responsive polymers and imaging agents (e.g. quantum dots, organic dyes or iron oxide nanoparticles) are attractive candidates for formulating theranostic systems, as they can be tracked following administration and provide controlled drug release in response to external stimuli. However, these systems are limited by non-biodegradability of polymers and by toxicity, photobleaching and possible leaching-out of the imaging agents 2. To overcome these limitations, biodegradable photoluminescent materials have been developed from diverse materials such as porous silicon nanostructures, conjugated polymeric nanoparticles containing π conjugated electron systems, and combinations of polyethylene glycol, citric acid, aliphatic diols and amino acid based polymers for *in vivo* biomedical applications such as tumor imaging and tracking the degradation progress of implanted scaffold materials 3-6. The degradability of these polymers and the superior photoluminescent properties such as high quantum yield, photobleaching resistance, and tunable emission up to near infrared region, make them unique for trackable drug delivery systems. Moreover, these polymers have demonstrated excellent compatibility and imaging ability both *in vitro* 7 and *in vivo* 5.

Thermo-responsive polymers have long been studied for drug delivery applications due to their attractive temperature-dependent drug release behavior. Poly(*N*-isopropylacrylamide) (PNIPAAm) and its copolymers are the most commonly used thermo-responsive polymers for such applications 8, 9. PNIPAAm undergoes a reversible phase-transition at a characteristic lower critical solution temperature (LCST) of 32°C 10-12. At temperatures below the LCST, the polymer becomes hydrophilic and swells by absorbing large quantities of water; whereas at temperatures higher than the LCST, the interactions among hydrophobic groups increase, causing the polymer to become hydrophobic by shrinking and releasing the payload. PNIPAAm is usually copolymerized with hydrophilic monomers such as acrylamide to increase the LCST above body temperature for controlled drug delivery applications as well as with monomers such as allylamine (AH) to provide amine functional groups for bioconjugation applications 13. Despite PNIPAAm's immense potential in the field of drug delivery and biomedical sciences, it suffers the disadvantage of non-degradability, which may cause inflammatory and toxic responses due to the prolonged presence of the polymer in the body 10, 11, 14. This limitation has provided the motivation for several research groups to develop biodegradable and temperature-sensitive PNIPAAm-based copolymers for drug delivery applications 15-17.

In the current work, we have developed a copolymer of PNIPAAm, AH, and a previously developed water soluble biodegradable photoluminescent polymer (WBPLP) 5 to form a novel thermo-responsive fluorescent polymer (TFP), and subsequently formulated the nanoparticles (TFP NPs). The unique combination of WBPLP and PNIPAAm may enable nanoparticle tracking *in vivo* using fluorescence imaging, while providing controlled and temperature-dependent release of the encapsulated payload. The presence of WBPLP in the copolymer may also elevate the LCST of the PNIPAAm due to added hydrophilic content in the polymer backbone, thus eliminating commonly used toxic acrylamide monomers to increase the LCST.

Another aspect of our work was the utilization of superparamagnetic iron oxide nanoparticles as core materials to coat the TFP polymer on. Iron oxide magnetic nanoparticles (MNPs) are usually 10-100 nm in size and by virtue of their magnetic property can be recruited at a specific site *in vivo.* The superparamagnetic nature of iron oxide nanoparticles makes them efficient T2 weighted magnetic resonance imaging (MRI) contrast agents that can be applied as imaging probes for early tumor detection 18. These attributes make MNPs potentially useful for tumor imaging as well as magnetic and image guided drug delivery 19, 20. This is highly beneficial in limiting the uptake of toxic chemotherapeutic molecules by healthy cells, thus improving drug delivery efficacy 21. Despite the immense potential of MNPs for tumor diagnosis and therapy, there remain a limited number of MNPs-based formulations approved for clinical use 22, possibly due to their reactive surface mediated toxicity. The proposed TFP polymer coating on iron oxide nanoparticles may help alleviate iron oxide toxicity and make an improved magnetic nanoparticle system. In addition, the TFP would provide fluorescent signals for optical imaging, making a multi-modal imaging system for enhanced imaging and diagnosis. Thus, we investigated TFP-coated MNPs (TFP-MNPs) core-shell system for potential magnetic targeting, multi-modal (optical and MRI) imaging, and simulated magnetic hyperthermia applications, so that both diagnosis and treatment of the disease is possible in a single setting.

## Materials and Methods

### Materials

All chemicals were purchased from Sigma-Aldrich (St. Louis, MO) and used without further modification. All cell lines were purchased from ATCC (Manassas, VA), and Dulbecco's modified Eagle's medium (DMEM), trypsin EDTA, fetal bovine serum (FBS), and penicillin-streptomycin were purchased from Invitrogen (Carlsbad, CA) and used for *in vitro* cell culture studies.

### Synthesis of fluorescent polymer

WBPLP was synthesized following our previously developed protocols 5. Briefly, equimolar ratios of citric acid and 1,8-octane diol were combined with L-cysteine, while keeping the L-cysteine to citric acid ratio at 0.8. The mixture was melted at 160°C for 20 minutes, subsequently cooled to 140°C and reacted for an additional 75 minutes to form BPLP-Cysteine oligomers. The BPLP-Cysteine oligomers were collected via precipitation using a water/1-4 dioxane mixture and were later freeze dried and subsequently combined with polyethylene glycol and amino acids to form water soluble BPLP (WBPLP).

### Copolymerization of WBPLP and AH

WBPLP was then conjugated with AH using carbodiimide chemistry 23. In brief, WBPLP (45 mg) was dissolved in 2-(N-morpholino) ethanesulphonic acid (MES) buffer (5 ml), followed by the addition of 1-Ethyl-3-(3-dimethylaminopropyl) carbodiimide (EDC) and *N*-Hydroxy Succinimide (NHS) (1:1). After 30 minutes of mixing on a rotator, AH (18.75 µl) was added and the reaction was continued for 12 hours at room temperature. The WBPLP-AH copolymer was then dialyzed using 500 Da molecular weight cut-off dialysis membranes (Spectrum Laboratories Inc, Rancho Dominguez, CA) for 24 hours to remove the unreacted chemicals.

### Synthesis of TFP NPs

To generate TFP NPs, WBPLP-AH was then copolymerized with *N*-isopropylacrylamide (NIPAAm) by radical polymerization in the presence of a crosslinker. In brief, the purified WBPLP-AH solution (5 ml) and NIPAAm (45 mg) were dissolved in deionized (DI) water (25 ml). Crosslinker, *N,N^'^*-Methylenebisacryamide (BIS, 5.85 mg) and surfactant, sodium dodecyl sulphate (SDS, 17.4 mg) were added to the mixture, while continuously stirring for 30 minutes. Ammonium persulphate (APS, 52.48 mg) and tetramethylethylenediamine (TEMED, 69 µl) were then added to initiate the radical polymerization, and the reaction was stirred for 4 hours under nitrogen at room temperature. The formed nanoparticle solution was dialyzed using 3500 Da molecular weight cut-off dialysis membranes for 24 hours to remove free surfactants and unreacted chemicals.

### Synthesis of TFP-MNPs

TFP-MNPs were synthesized using silane-functionalized MNPs (silane-MNPs) as templates for TFP conjugation. Silane-MNPs were synthesized by dispersing MNPs in 99% ethanol by sonication (50 W, 5 minutes), followed by the addition of acetic acid (2 ml). After 5 minutes of further sonication, the reaction was transferred to a stir plate and vinyltrimethoxysilane (0.49 ml) was added. The reaction was carried out for 24 hours at room temperature. Silane-MNPs were then washed several times with 99% ethanol and collected using a magnet. To prepare the TFP-MNPs, silane-MNPs (10 mg) were sonicated in DI water (25 ml) at 50 W for 10 minutes. The purified WBPLP-AH (100 mg), NIPAAm (45 mg), BIS (5.85 mg) and SDS (17.4 mg) were added to the reaction while sonicating. The reaction was then transferred to a stir plate, and APS (52.48 mg) and TEMED (69 µl) were added to the reaction with vigorous stirring. The reaction was carried out under nitrogen for 4 hours at room temperature. The TFP-MNPs were collected by a magnet and washed several times with DI water to remove surfactants and unreacted chemicals.

### Physicochemical characterization

Particle size, morphology, polydispersity index (PDI), and surface charge (ζ potential) were evaluated using transmission electron microscopy (TEM; Technai, JEOL 1200 EX, Tokyo, Japan) and dynamic light scattering (DLS; ZetaPals, Brookhaven Instrument, Holtsville, NY). To prepare samples for TEM, a drop of nanoparticle solution was put on the surface of a Formvar coated 200-mesh copper grid (Electron Microscopy Sciences, Hatfield, PA) and air-dried before loading onto a microscope. For DLS, 1 mg/ml nanoparticle solution was directly added to a cuvette for size, polydispersity and surface charge measurements. Next, Fourier transform infrared spectroscopy (FTIR; Nicolet 6700 FT-IR spectrometer, Thermo Fisher Scientific, Madison, WI) was performed to analyze characteristic peaks associated with the chemical bonds in the polymer chain. All FTIR samples were purified, lyophilized powders. The LCST of the TFP NPs was also determined quantitatively by heating the nanoparticle solution in 1°C increments from room temperature up to 45°C in a glass cuvette, and by measuring the absorbance of the nanoparticle solution at 500 nm wavelength using UV-Vis spectrophotometry (Biorad, SmartSpec Plus, Life Science Research, Hercules, CA).

Nanoparticle stability and degradation were studied at physiological conditions. To study particle stability, the TFP-MNPs were incubated in phosphate buffered saline (PBS) or 10% FBS solution at 37°C and the particle size was measured up to 72 hours using DLS. To study the degradation of the TFP shell, TFP-MNPs were suspended in DI water and incubated at 37°C over a period of time. At each time point, nanoparticles were collected by a magnet, and dry weight of the nanoparticles was recorded. A relative percentage of dry weights of the nanoparticles at all the time points were calculated with respect to the initial dry weight of the nanoparticles.

### Magnetic characterization

The iron content in the nanoparticles was evaluated using iron assays as described previously 24. Briefly, 100 µl of the nanoparticle solution was incubated with 50% (v/v) hydrochloric acid at 37°C overnight. Subsequently, APS solution (1 mg/ml) and 0.1 M potassium thiocyanate solution were added with 15 minutes incubation between each step. The absorbance readings of the samples were taken at 520 nm using a UV-Vis spectrophotometer (Infinite M200 plate reader, Tecan, Durham, NC). Additionally, magnetic recruitment of the TFP-MNPs were tested in the presence of an external magnet and compared with the TFP-MNPs suspension in the absence of the external magnet.

The magnetic properties of TFP-MNPs were analyzed using a vibrating sample magnetometer (VSM, KLA-Tencor EV7, San Jose, CA) and compared to those of bare MNPs. The samples consisting of equal amounts of iron were embedded in wax and hysteresis loops were obtained by varying the magnetic fields at room temperature. Then MR imaging was done on agarose phantoms containing varying concentrations of TFP-MNPs (0, 0.25, 0.5, 1 and 2 mg/ml). Agarose phantoms containing TFP NPs (without MNPs) were imaged as negative control. T2 weighted images were acquired using TR: 2500 ms, TE: 10 ms, FOV: 40x40 mm, and with a slice thickness of 1 mm.

### Fluorescence characterization

The fluorescence from the nanoparticles was observed in UV light. The positive control contained WBPLP solution and the negative control contained PNIPAAm-AH solution. Furthermore, the effect of temperature on the fluorescence intensity of the nanoparticles was investigated. Briefly, the TFP NPs (3 mg/ml) in a tube were immersed in a water tank that was heated using a temperature controller. The sample was excited with a blue laser (473 nm) and the emitted light was passed through a 532 nm long pass filter. The fluorescence intensity was recorded as voltage read out from a high-speed digital oscilloscope. The measurements were taken at temperatures ranging from 25°C to 45°C with an increase of 0.5°C. The fluorescence intensities of these measurements were then converted to the percentage lost in the fluorescence intensity as a function of temperature.

### Drug loading and release

For drug loading and release studies, hydrophilic doxorubicin hydrochloride (Dox; Tocris Bioscience, Ellisville, MO) was used as an anti-cancer drug model. Freeze-dried TFP-MNPs (10 mg) were suspended in Dox (0.02% w/v in PBS) solution and incubated at 4°C for 3 days, with gentle stirring to allow loading of the drug into the nanoparticles by virtue of the drug absorption via polymer swelling at low temperatures. After 3 days, the Dox-loaded TFP-MNPs (Dox-TFP-MNPs) were separated using a magnet (1.3 T) and the supernatant was collected to determine the Dox encapsulation efficiency using the following formula:


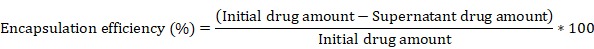


To study thermo-responsive drug release, the Dox-TFP-MNPs were incubated at 25°C (room temperature, < LCST), 37°C (physiological temperature, < LCST), or 41°C (> LCST) in PBS. At predetermined time points, an external magnet was used to separate the nanoparticles in each of the three groups, and 1 ml of the supernatant was collected. The same volume was replenished with fresh PBS. Dox concentrations in the collected samples were measured by UV-Vis spectrofluorometer at 470 nm excitation and 585 nm emission wavelengths and calculated using a Dox standard curve.

### *In vitro* cell studies

For cytocompatibility studies, human dermal fibroblasts (HDFs) and normal prostate epithelial cells (PZ-HPV-7) were seeded in a 96-well plate at a density of 5000 cells/well and maintained at 37°C and 5% CO_2_ for 24 hours. The culture medium was replaced with media containing increasing concentrations of TFP-MNPs (0, 50, 100, 200, 300, and 500 µg/ml) and the cells were incubated for 12 and 24 hours. Cells exposed to media only served as positive controls. The cell viability was determined at each time point using cell viability MTS assays (CellTiter 96 Aqueous One Solution Cell Proliferation Assay, Promega, Madison, WI) following the manufacturer's instructions.

Cellular uptake of nanoparticles was studied on A431 and G361 skin cancer cells as well as on PC3 and LNCaP prostate cancer cells. First, the cells were seeded in a 96-well plate as described earlier. Following overnight incubation at 37°C, the media was replaced with different concentrations of TFP-MNPs (0, 50, 100, 200, 300, and 500 µg/ml) prepared in RPMI-1640 and the well plate was incubated for 2 hours. Cells that were not exposed to nanoparticles served as controls. Following incubation, the cells were washed with PBS three times and then lysed using 1X Triton. The contents in the wells were then analyzed using iron assay and Picogreen DNA assay (Invitrogen, Carlsbad, CA) to quantify the nanoparticle amount and cell protein content per well.

For cancer cell killing studies, skin cancer cells (A431, G361) and prostate cancer cells (PC3) were seeded at a density of 5000 cells/well in 96-well plates and incubated overnight at 37°C to facilitate cell attachment. The following day, the cell media was aspirated, and the cells were treated with media only, free Dox, Dox-TFP-MNPs or empty TFP-MNPs. Free Dox was dosed at the IC_50_ value of the drug with respect to each of the cell lines (37 nM for A431, 32 nM for G361, and 258 nM for PC3 cells). The dose of Dox-TFP-MNPs was then calculated as the concentration of nanoparticles which would release a cumulative IC_50_ dose of Dox in 24 hours. The empty TFP-MNPs were dosed at the same concentration as Dox-TFP-MNPs. The treated cells were incubated at 37°C or 41°C for 24 hours. The cell viability was then quantified by MTS assays.

### *In vivo* animal studies

All *in vivo* studies were conducted in compliance with the guidelines set by the University of Texas at Arlington and the University of Texas Southwestern Institutional Animal Care and Use Committees. NOD SCID mice (6-8 weeks old males) purchased from the University of Texas Southwestern mouse breeding core were used for *in vivo* studies. Prostate cancer xenograft models were developed by injecting subcutaneously into both flanks of the animal, DAB2 interactive protein knockdown (KD) prostate cancer cells (PC3-KD) (~5x10^5^ cells/site), as described elsewhere 25. The mice were observed periodically, and the experiments were performed when the tumors were palpable.

For fluorescence imaging, BPLP-MNPs (100 μl of 5 mg Fe/kg), TFP-MNPs (100 μl of 5 mg Fe/kg), or TFP NPs (100 μl of 3 mg/ml) were injected intra-tumorally. BPLP-MNPs were used as control nanoparticles and were synthesized as described elsewhere 7. The flank tumors were then imaged using an *in vivo* Kodak imaging system (Carestream Health Inc., Rochester, NY). The relative fluorescence intensity from the tumors injected with nanoparticles was calculated by subtracting the fluorescence intensity of the tumors that were not injected with the nanoparticles.

For MRI studies, a baseline MRI was obtained on flank tumors by T2 weighted imaging (TR = 2500 ms, TEeff = 60 ms, FOV = 40x40 mm, slice thickness = 1 mm). Then saline and TFP-MNPs (1 mg) with or without magnetic targeting were injected via the tail vein and MR images were taken 24 hours later. The difference in MRI signal intensity between the groups was then analyzed using Image J.

For *in vivo* therapeutic efficacy study, C57BL6 mice were injected with B16F10 skin cancer cells in the flanks. When the tumors grew 8-10 mm^3^ in volume, saline, free Dox (40 µg/ml, 200 µl), empty TFP-MNPs (0.8 mg/ml, 200 µl) or Dox-TFP-MNPs (0.8 mg/ml, 200 µl) were intravenously injected. The animals were then placed on a heating pad at 37°C for 30 min with a 1.3 T magnet placed near the tumors to magnetically recruit nanoparticles in the region. The animals were then allowed to return to their cages. At each predetermined time point, the tumor volumes were measured using Vernier caliper. On day 15, the surviving animals were sacrificed and the tumor volumes were recorded.

### Statistical analysis

The results obtained were analyzed using one-way analysis of variance with *p* < 0.05 and post hoc comparisons. All the experiments were repeated multiple (at least two) times with a sample size of four (n=4). All the results were presented as mean ± standard deviation if not specified.

## Results

### Physicochemical characterization

**Figure 1:**
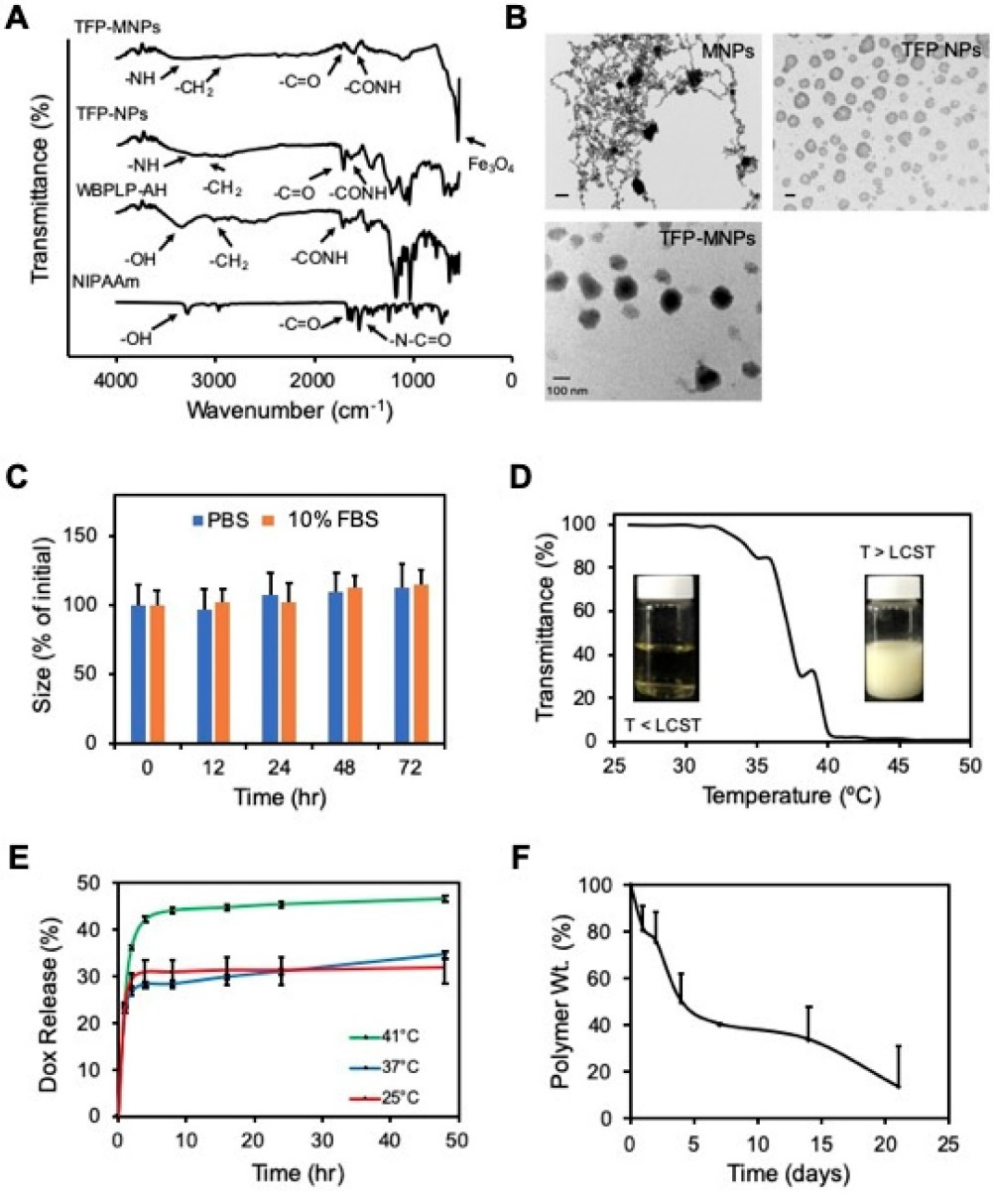
** Physicochemical characterization of nanoparticles.** (A) FTIR spectra of TFP-MNPs, TFP NPs, WBPLP-AH and NIPAAm with arrows indicating the peaks associated with bonds in polymer backbone and MNPs. (B) TEM images of MNPs, TFP NPs, and TFP-MNPs (all scale bars = 100 nm). (C) Stability of TFP-MNPs in PBS and 10% FBS at 37°C for 72 hours as measured by changes in nanoparticle size. (D) Phase transition of TFP NPs at LCST (39°C). (E) Temperature-dependent Dox release kinetics showing higher release at 41°C compared to 37°C and 25°C. (F) Degradation profile of TFP shell on MNPs core showing 86% polymer weight loss in 13 days.

The copolymerization of WBPLP-AH and PNIPAAM, and their incorporation into the TFP NPs and TFP-MNPs was confirmed via FTIR analysis (Figure 1A). The FTIR spectrum of NIPAAm showed characteristic peaks corresponding to C=O (1730 cm^-1^) and N-C=O (1590 cm^-1^) bonds. WBPLP-AH spectrum had peaks corresponding to CH_2_ (3015 cm^-1^), -OH stretching (3496.2 cm^-1^) and CO-NH (1704 cm^-1^) bond, which confirmed the successful conjugation of AH to the WBPLP. The FTIR spectrum of TFP NPs retained the peaks corresponding to CH_2_ groups (3010 cm^-1^) from WBPLP-AH and CO-NH (1635 cm^-1^) and C=O (1705 cm^-1^) bonds from NIPAAm. The TFP-MNPs spectrum also showed the presence of these bonds in addition to the Fe_3_O_4_ (540 cm^-1^) peak, confirming the incorporation of MNPs. These findings were in agreement with our previous observations confirming the presence of MNPs 10 and all the corresponding bonds from WBPLP and PNIPAAm coatings 5, 26.

Hydrodynamic diameter, PDI and ζ potential of the TFP NPs was ~150 nm, 0.28 and -13.4 mV, respectively while that of TFP-MNPs was ~135 nm, 0.07 and -31.0 mV, respectively (Table 1). The TEM images showed MNPs as dark circular particles and the TFP NPs appeared as lighter polymeric particles (Figure 1B). The TFP-MNPs showed a dark magnetic core and a lighter polymeric shell in their structure. Stability studies indicate that the TFP-MNPs varied by less than 15 % of their original size in PBS and 10% FBS solution at 37°C over a period of 72 hours (Figure 1C). These results indicate that the nanoparticles are stable and would likely not aggregate under physiological conditions. Furthermore, the phase transition behavior of TFP NPs was characterized by measuring absorbance (λ_max_ = 500 nm) of the nanoparticle solutions at each unit increment in temperature using a UV-Vis spectrophotometer. The LCST of the nanoparticles (39°C) was evaluated at the solution temperature where the transmittance drops to 50% of initial value (Figure 1D). At temperatures below LCST, the polymer was hydrophilic, making the solution clear. The solution turned turbid when the polymer became hydrophobic at temperatures equal to or greater than the LCST. This result confirms the thermo-responsive behavior of TFP NPs even after copolymerization of PNIPAAm with AH and WBPLP.

Dox loading efficiency in TFP-MNPs was ~90%, which was higher in comparison to the Dox loading efficacy in previously synthesized PNIPAAm-AAm-AH-based MNPs (82%) by our group. The higher loading of Dox in TFP-MNPs might be due to either the interaction between Dox and TFP or more polymeric swelling of TFP at temperatures below the LCST while loading Dox. A temperature-dependent biphasic Dox release was observed (Figure 1E). Dox was released in a significantly higher amount at 41°C (temperature > LCST of TFP) compared to that of 37°C and 25°C. There was no difference between Dox release at 37°C and 25°C, as both the temperatures were below the LCST of TFP. Since drug release can also be caused by degradation of the polymer shell, degradation of the TFP coating on MNPs in DI water was studied over time. It was observed that the TFP-MNPs lost 31% polymer weight during the first 4 days (Figure 1F). The degradation rate was then reduced, which resulted in 37% polymer weight loss over 13 days. The reduction in the degradation rate was due to the presence of PNIPAAm and AH slowing down the hydrolysis of the WBPLP. It is speculated that the TFP degradation will take longer than that of WBPLP alone.

### Magnetic characterization

**Figure 2:**
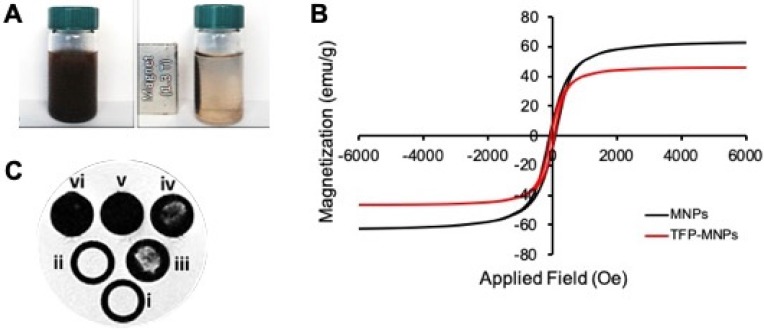
** Magnetic properties of nanoparticles. (A)** Photographs showing TFP-MNPs suspension in water (left) and recruitment of TFP-MNPs towards a 1.3 T magnet (right). **(B)** Hysteresis loops of bare MNPs and TFP-MNPs followed the same trend demonstrating their superparamagnetic behavior. **(C)** T2 weighted MR images of agarose phantoms containing i. agarose only, ii. TFP NPs, and iii-vi. TFP-MNPs at 0.25, 0.5, 1, and 2 mg/ml concentrations, respectively, showing increasing negative contrast with increasing iron oxide concentration in agarose phantoms.

The TFP-MNPs were comprised of approximately 75% mass of iron (Table 1). Moreover, in the absence of a magnet, nanoparticles were suspended and well-dispersed in DI water (Figure 2A). While in the presence of a 1.3 T magnet, nanoparticles concentrated toward the magnet, demonstrating the recruitment of nanoparticles via magnetic targeting. VSM results indicate that the iron oxide within TFP-MNPs retained their superparamagnetic properties and showed a similar hysteresis loop as bare MNPs (Figure 2B). The saturation magnetization for bare MNPs and TFP-MNPs was 63 and 46 emu/g, respectively. A remanence and coercivity of 6.24 (M_r_/M_s_) and 59.9 Oe, respectively, was quantified for the TFP-MNPs, while bare MNPs had a remanence of 8.16 (M_r_/M_s_) and coercivity of 75.5 Oe. In addition, agarose phantoms containing these particles produced a distinct negative contrast in MRI with greater negative contrast observed with increasing concentration of the particles (Figure 2C). These observations show that the TFP-MNPs possess strong magnetic properties as well as can potentially act as MRI contrast agents.

### Fluorescence properties

**Figure 3:**
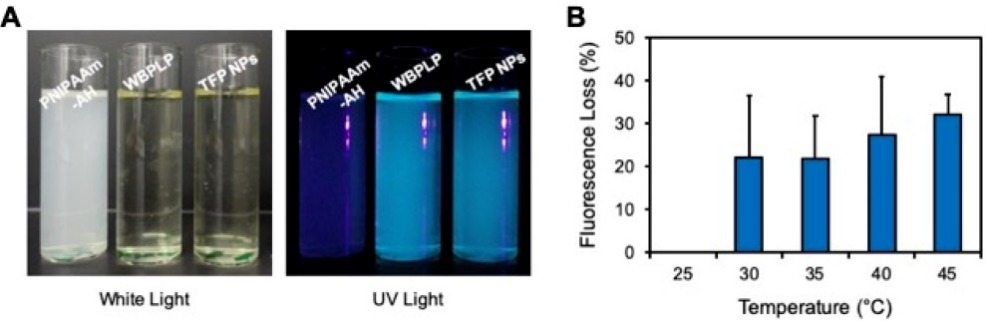
** Characterization of fluorescence property.** (A) Photographs of PNIPAAm-AH, WBPLP and TFP NPs samples in ambient white light and UV light. (B) Fluorescence intensity of TFP NPs was measured at increasing temperatures and plotted as fluorescence intensity lost as a function of temperature.

Samples of PNIPAAm-AH, WBPLP, and TFP NPs were subjected to natural light and UV illumination in dark. There was no fluorescence from any of the samples in white light (Figure 3A). However, a bright fluorescence was observed from WBPLP and TFP nanoparticles under UV illumination. The negative control PNIPAAm-AH did not display any fluorescence contrary to TFP NPs, which demonstrated fluorescence under UV illumination, suggesting the origins of TFP NP fluorescence from WBPLP. Furthermore, a temperature-dependent fluorescence study was performed. The mean fluorescence intensity of TFP remained the same with increasing temperature (Figure 3B). The fluorescence photobleaching effect was also tested at 23°C and 46°C. Even after more than 10 minutes of continuous laser excitation, no photobleaching was observed and the fluorescence intensity remained stable. This indicates that the fluorescence of the TFP-MNPs will not be affected by temperature changes.

### *In vitro* cell studies

**Figure 4:**
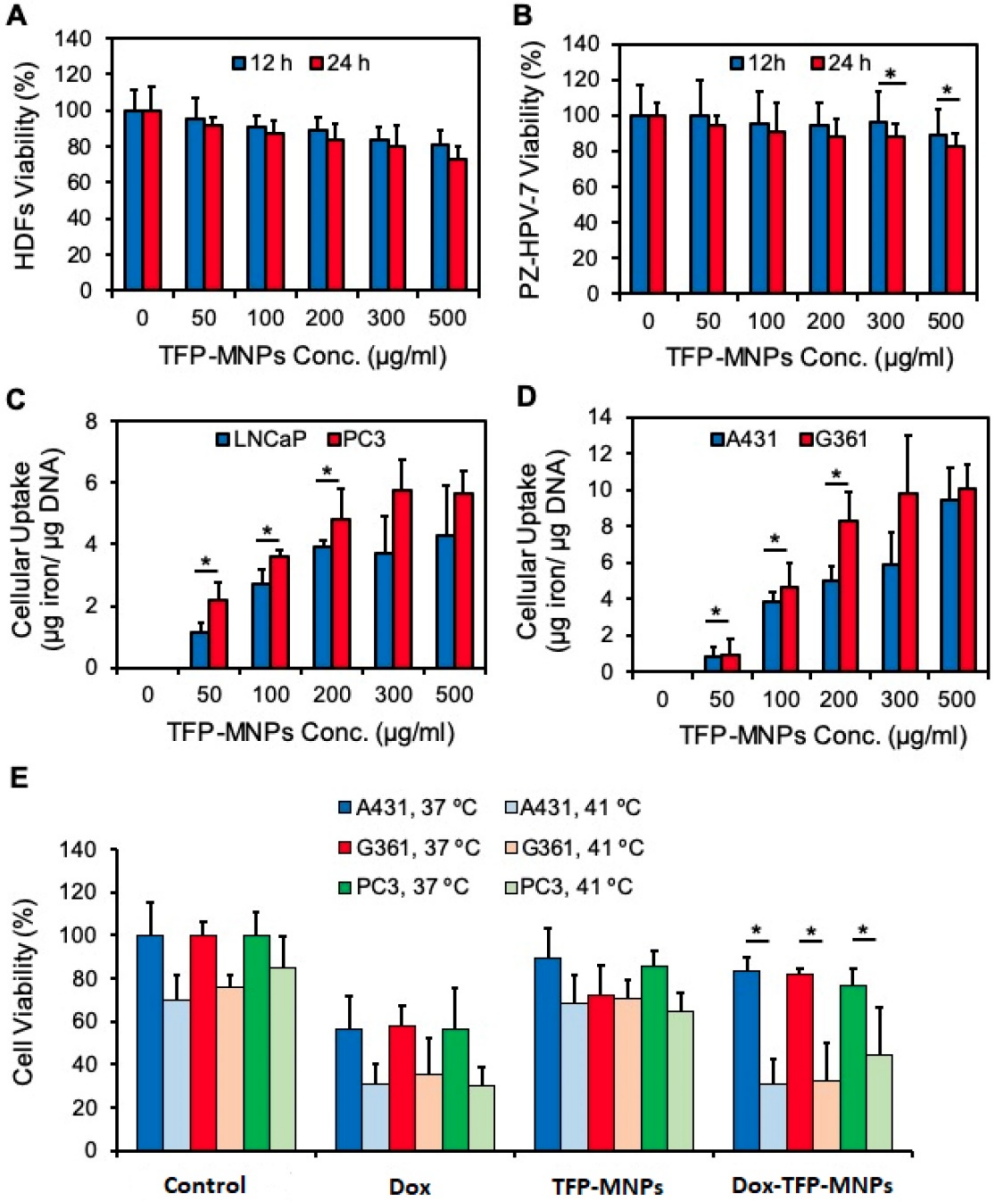
***In vitro* cytocompatibility, cellular uptake, and therapeutic efficacy of nanoparticles**. (A) Cytocompatibility profiles of TFP-MNPs in normal human dermal fibroblasts (HDFs) and (B) normal prostate epithelial cells (PZ-HPV-7) as measured by MTS assays. (C) Cellular uptake profiles of TFP-MNPs on LNCaP and PC3 prostate cancer cells and (D) A431 and G360 skin cancer cells. (E) Cytotoxicity of nanoparticles on skin cancer (A431, G361) and prostate cancer (PC3) cells at 37°C and 41°C as measured by MTS assays. *p < 0.05.

Cytocompatibility of TFP-MNPs was tested on HDFs and PZ-HPV-7 cells. It was observed that the TFP-MNPs were not toxic at the tested concentrations for both cell types after 12 hours of incubation (Figure 4A-B). However, after 24 hours of exposure, the TFP-MNPs showed some level of toxicity towards HDFs, 27% cell death at 500 µg/ml concentration compared to the controls. The particles were more compatible with the PZ-HPV-7 cells, about 12% and 18% cell death was observed at 300 and 500 µg/ml concentrations, respectively. PZ-HPV-7 cell viability was above 80% at all concentrations of TFP-MNPs.

The uptake of TFP-MNPs by A431 and G361 skin cancer cells and LNCaP and PC3 prostate cancer cells was investigated. A dose-dependent uptake of the particles was observed in all cancer cell lines following 2 hours of incubation with the particles (Figure 4C-D). Higher uptake of TFP-MNPs was seen in skin cancer cells especially at higher concentrations. Furthermore, uptake seemed to saturate between 200-300 µg/ml TFP-MNPs concentration in the case of all the cell lines.

A cancer cell killing study was performed to determine the *in vitro* therapeutic efficacy of Dox-TFP-MNPs with changes in temperature. It could be seen clearly that in the case of all the cell lines (A431, G361, PC3) treated with Dox-TFP-MNPs, greater cell death was observed at 41°C (about 31%, 32% and 44% cell viability, respectively) compared to the cell death at 37°C (about 83%, 82% and 77% cell viability, respectively) (Figure 4E). About 30% of the cell death at 41°C could be attributed to hyperthermia as seen in the case of the control groups at 41°C. Minimal cell death was observed on exposure of the cell lines to empty TFP-MNPs.

### *In vivo* imaging and therapeutic efficacy

**Figure 5:**
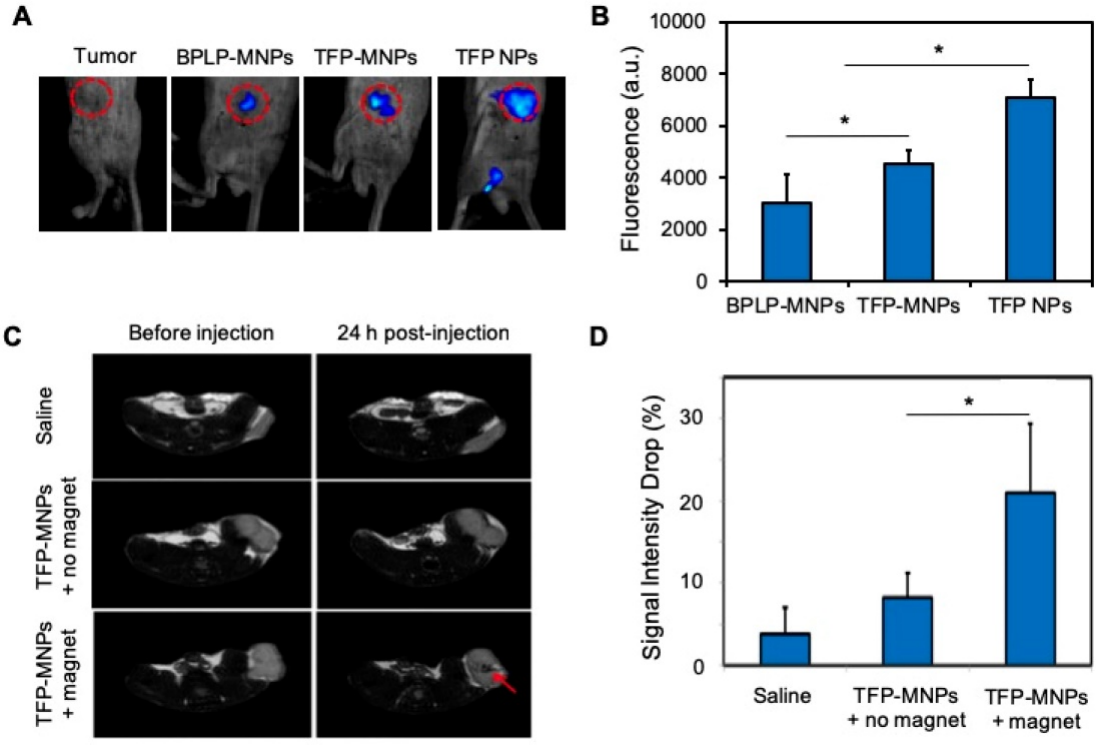
***In vivo* imaging and magnetic targeting of nanoparticles**. (A) Fluorescent images of prostate cancer tumors in mice after intra-tumoral injection of nanoparticles. (B) Relative fluorescence intensities from the panel A (*p < 0.05). (C) T2-weighted MR images of prostate tumors in mice before and 24 hours after i.v. injection of nanoparticles in the presence or absence of an external magnet. The red arrow indicates negative contrast generated due to accumulation of TFP-MNPs. (D) MRI signal intensity drop in prostate cancer tumors 24 hours post-injection from the panel C (*p < 0.05).

*In vivo* fluorescence imaging was performed after intra-tumoral injections of nanoparticles. The control tumors (without nanoparticle injections) did not display any fluorescence (Figure 5A). A bright fluorescence was detected from the tumors injected with TFP NPs, which was then reduced significantly for TFP-MNPs due to the presence of darker MNPs (Figure 5B). However, the fluorescence intensity from TFP-MNPs was significantly higher than that of hydrophobic BPLP-MNPs. These results show that the TFP-MNPs can overcome the limitation of reduced fluorescence from our previously developed BPLP-MNPs 7.

### Magnetic targeting and *in vivo* MR imaging

Tumor bearing mice were injected with TFP-MNPs via tail vein injections and the localization of the nanoparticles was studied in the presence or absence of a 1.3 T magnetic field. The tumors in both the magnetic and non-magnetic field treated group were imaged before and 24 hours post injection. A negative contrast was observed in the tumors of mice injected with TFP-MNPs and subsequently treated with a localized magnetic field, indicating that the particles were able to be recruited at the tumor site (Figure 5C). TFP-MNPs administered animals without the localized magnetic field treatment did not have any significant darkening in the tumor region. Analysis of the signal intensity values of the tumors showed a significant drop (~21%) in signal intensity in animals injected with TFP-MNPs in the presence of localized magnetic fields (Figure 5D). The controls and the groups treated with TFP-MNPs in the absence of magnetic targeting showed signal intensity drops of 3.8% and 8.2%, respectively.

### *In vivo* therapeutic efficacy

**Figure 6:**
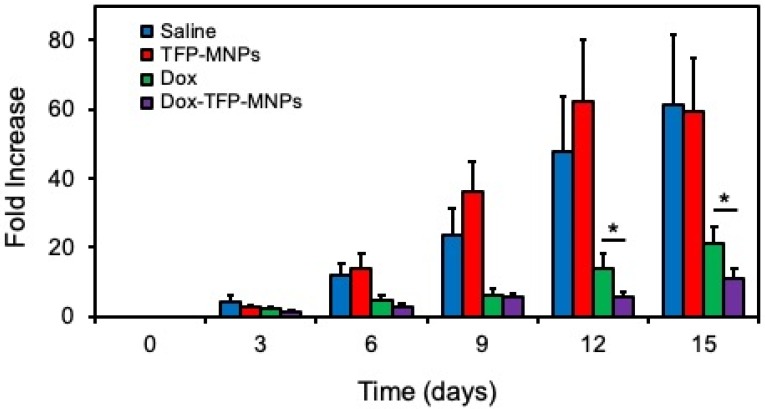
***In vivo* therapeutic efficacy of nanoparticles**. Skin cancer B16F10 tumor volume fold increase in mice after i.v. injection of nanoparticles in the presence of 1.3 T external magnet (*p < 0.05).

*In vivo* therapeutic efficacy of Dox-TFP-MNPs was studied in animals implanted with B16F10 skin tumors in the presence of a 1.3 T magnetic field. At the end of the 15-day study, only an 11-fold increase in the original tumor volume was observed in animals treated with Dox-TFP-MNPs in the presence of the magnetic field (Figure 6). On the other hand, the control animals given saline injections showed a 61-fold increase in their original tumor volumes. The animals in the empty TFP-MNPs and free Dox group showed 59-fold and 21-fold increases in their tumor volumes, respectively.

## Discussion

Advancements in cancer diagnosis, treatment and preventative approaches have led to a drop in cancer mortality rates, showing marginal improvements in survival. Although chemotherapy has been successful in reducing cancer related deaths, it often has poor pharmacokinetic profiles in addition to nonspecific toxicity. Nanoparticle mediated drug delivery of chemotherapeutics potentially seeks to eliminate these limitations and have improved survival outcomes with many clinically developed nanoparticulate systems demonstrating reduced systemic toxicity, albeit they have yet to succeed in improving long-term survival. This warrants the development of newer nanoscale systems with improved functionalities and additions of multifunctionality into the nanoparticle design. With the goal to develop a degradable PNIPAAm-based theranostic drug delivery system, we investigated the synthesis and characterization of biodegradable TFP NPs and a core-shell system containing a magnetic core and TFP shell (TFP-MNPs).

Physicochemical characterization of our nanoparticles showed well formulated nanoparticles with excellent colloidal stability in physiological conditions. The negative surface charges of the TFP-MNPs potentially repel negatively charged albumin molecules in the serum, preventing agglutination mediated aggregation 27. Furthermore, TFP holds the unique characteristics of thermo-responsiveness from PNIPAAm as well as fluorescence and degradability from WBPLP. The increase in LCST (39°C) of the copolymeric nanoparticles compared to PNIPAAm nanoparticles (LCST = ⁓32-34°C) can be attributed to the incorporation of hydrophilic WBPLP-AH 11, 15. The sharp volume phase transition of the nanoparticles is advantageous, enabling a burst release of drugs followed by a more sustained release, especially at 41°C. The degradation of the polymer shell was mainly due to hydrolysis of WBPLP, eventually breaking down PNIPAAm into smaller fragments that can be potentially cleared *in vivo*. Interestingly, the fluorescence from WBPLP was well preserved in the new copolymer even at higher temperatures. In addition, incorporating MNPs into the TFP NPs has added advantages of magnetic targeting as well as using these particles as MRI contrast agents. TFP coating did not affect the superparamagnetic behavior of MNPs. A small decrease in the saturation magnetization of TFP-MNPs compared to bare MNPs can be attributed to the diamagnetic moment of the polymeric coating 23. Similar PNIPAAm copolymeric systems grafted on MNPs have been shown to have a slight decrease in saturation magnetization, which has been attributed to the grafting of polymers to the iron oxide core 28. The presence of an iron oxide magnetic core in TFP-MNPs renders them the ability to dephase proton spins causing a reduction in MR signal intensity, making them useful as T2 contrast agents 29.

Overall, *in vitro* data including cell studies further highlights the potential of TFP-MNPs as a drug delivery vehicle for solid tumors. Surface properties of nanoparticles play a major role in cellular toxicity and uptake. The negatively charged polymer shell is potentially alleviating MNP-mediated toxicity 30, 31 by preventing MNP interactions with the cell membrane. Further, the differential uptake of TFP-MNPs in prostate and skin cancer cells is in agreement with our previous studies on the uptake of BPLP-MNPs and WBPLP-MNPs in PC3 and LNCaP cell lines where the more hydrophilic WBPLP-MNPs showed significant preferential uptake into PC3 cell lines relative to LNCaP cell lines 7. It has been shown that MNPs coated with proteins exhibited preferential cellular uptake in different cancer cell lines, even in isogenic cell lines 32. The preferential uptake of TFP-MNPs by PC3 and G361 cancer cell lines, which have higher metastatic potential than LNCaP and A431 cell lines, indicates the utility of TFP-MNPs to target more aggressive kinds of cancers 33. Further, Dox-TFP-MNPs were effective in killing cancer cells under simulated hyperthermia conditions. The insignificant difference in cell killing between free Dox and Dox-TFP-MNPs at 41°C across all cell lines was potentially due to the fact that free Dox experiences increased cellular uptake at temperatures exceeding 40°C as well as has the ability to be actively cytotoxic without entering cells 34, 35. The cell death in untreated controls and empty TFP-MNPs group at 41°C was attributed to the negative effects of hyperthermia 36, 37.

Our preliminary *in vivo* data showed the dual-imaging, magnetic targeting, and therapeutic efficacy potential of the TFP-MNPs. A significantly higher fluorescent intensity in TFP NPs treated groups compared to TFP-MNPs was possibly due to the presence of a darker iron oxide core, which has been shown to quench fluorescence on contact with various fluorescent materials 38-40. The quenching of fluorescence was quantitatively lesser in TFP-MNPs compared to MNPs coated with the fluorescent hydrophobic BPLP polymer. We believe this difference is due to the nature of the polymer interaction with the MNP surface. The covalent bonding of TFP to the MNPs holds the polymer shell in place compared to the physical adsorption of BPLP on the MNP surface, increasing the possibility of BPLP shell/fragments separating from MNPs. Further, magnetic targeting played a key role in recruiting TFP-MNPs at the tumor site in sufficient doses to be therapeutically effective. The use of magnetic targeting to deliver drugs to tumors relies on the physical force of magnetic fields to partition the magnetic carrier from the arteriole wall into the tumor region resulting in increased drug localization and retention even after the subsequent removal of the applied magnetic field 18, 41. Similar drug carrying magnetic nanocarriers using magnetic targeting have been shown effective in delivering various agents like mitoxantrone, epirubicin and doxorubicin in tumor bearing rats and cause tumor remission in various cancer models 42-44.

Our study has a few limitations. We have used different cell lines from two different cancer types for *in vitro* experiments and two different animal models for *in vivo* experiments. While our TFP-MNPs are not tested extensively on one particular disease, we intended to show the theranostic feasibility and effectiveness of our nanoparticles on different solid tumor types. In addition, in the current age of active (receptor-mediated) targeting of nanoparticles, our nanoparticle design lacks this advantageous element. In the future, we plan to conjugate TFP-MNPs with targeting moieties and cell penetrating peptides, giving the nanoparticle system specificity towards solid tumors and explore a targeted site-specific delivery. Nonetheless, we could show the effectiveness of TFP-MNPs in controlling tumor growth by using magnetic targeting as a standalone approach. Our results demonstrated a great potential of TFP-MNPs as a theranostic platform technology. The development of TFP-MNPs will not only overcome the drawbacks of the long-term toxicity of quantum dots and poor photostability of organic dyes, but also allow simultaneous diagnosis and treatment of cancers in a single setting. In the future, these nanoparticles with the use of alternating magnetic fields, could be used for producing heat for hyperthermia therapy and temperature-controlled drug release, enabling combinational treatment options 23, 24. Therefore, we think that TFP-MNPs can serve as an effective platform for future development of theranostic nanoparticles.

## Conclusion

We developed a novel TFP and its core-shell structure with magnetic nanoparticles - TFP-MNPs. TFP-MNPs maintained excellent colloidal stability and had a degradable shell that eliminates long-term toxicity concerns and bypasses the size limitations for *in vivo* clearance in the traditional nanoparticle designs. The thermo-responsiveness of the PNIPAAm-AH was preserved after copolymerization with WBPLP to form TFP. The imaging studies showed optical and MR imaging capabilities using TFP-MNPs. Finally, the cytocompatible nanoparticles magnetically recruited to the tumor region delivered therapeutic doses of doxorubicin to inhibit tumor growth. We showed multifunctional capabilities of TFP-MNPs including multimodal imaging, magnetic targeting, and thermo-responsive drug release, making TFP-MNPs an excellent platform of theranostic systems for future cancer diagnosis and therapy.

## Figures and Tables

**Table 1:** Physicochemical characterization of nanoparticles

Sample	Diameter (nm)	PDI	ζ Potential (mV)	Iron (%)
MNPs	10^a^	0.30	-5.1	100
Silane-MNPs	18^b^	0.35	-21.0	__
TFP NPs	150	0.28	-13.4	__
TFP-MNPs	135	0.07	-31.0	75

^a^Size provided by the supplier ^b^Size obtained from TEM analysis (image not shown)

## Abbreviations

A431: Skin cancer cell line
AH: Allylamine
APS: Ammonium persulphate
BIS: N,N'-Methylenebisacryamide
Da: Dalton
DI: deionized
DLS: Dynamic Light Scattering
DMEM: Dulbecco's modified Eagle's medium
EDTA: Ethylenediaminetetraacetic acid
FBS: Fetal bovine serum
FTIR: Fourier Transform Infrared Spectroscopy
G631: Skin cancer cell line
HDFs: Human Dermal Fibroblasts
LCST: Lower critical solution temperature
LNCaP: Prostate cancer cell line
MES: (N-morpholino) ethanesulphonic acid
MNPs: Magnetic nanoparticles
PBS: Phosphate buffered saline
PC3-KD: DAB2 interactive protein knockdown (KD) prostate cancer cells
PC3: Prostate cancer cell line
PNIPAAm: Poly(*N*-isopropylacrylamide)
PZ-HPV-7: Prostate epithelial cells
TFP: Temperature sensitive fluorescent polymer
SDS: Sodium dodecyl sulphate
TEM: Transmission electron microscopy
TEMED: Tetramethylethylenediamine
UV-Vis: Ultra violet visible
VSM: Vibration sample magnetometry
WBPLP: Water soluble biodegradable photoluminescent polymer
